# Circus Activities as a Health Intervention for Children, Youth, and Adolescents: A Scoping Review

**DOI:** 10.3390/jcm12052046

**Published:** 2023-03-04

**Authors:** Free Coulston, Kate L. Cameron, Kath Sellick, Madeline Cavallaro, Alicia Spittle, Rachel Toovey

**Affiliations:** 1 Faculty of Medicine, Dentistry and Health Sciences, The University of Melbourne, Melbourne 3052, Australia; 2 Clinical Sciences, Murdoch Children’s Research Institute, Melbourne 3052, Australia; 3 Physiotherapy Department, St Vincent’s Hospital, Melbourne 3065, Australia; 4 Newborn Services, The Royal Women’s Hospital, Melbourne 3052, Australia

**Keywords:** children, circus, paediatrics, scoping review, health outcomes, social-emotional health, physical health

## Abstract

Circus activities are emerging as an engaging and unique health intervention. This scoping review summarises the evidence on this topic for children and young people aged up to 24 years to map (a) participant characteristics, (b) intervention characteristics, (c) health and wellbeing outcomes, and (d) to identify evidence gaps. Using scoping review methodology, a systematic search of five databases and Google Scholar was conducted up to August 2022 for peer-reviewed and grey literature. Fifty-seven of 897 sources of evidence were included (42 unique interventions). Most interventions were undertaken with school-aged participants; however, four studies included participants with age ranges over 15 years. Interventions targeted both general populations and those with defined biopsychosocial challenges (e.g., cerebral palsy, mental illness, or homelessness). Most interventions utilised three or more circus disciplines and were undertaken in naturalistic leisure settings. Dosage could be calculated for 15 of the 42 interventions (range one-96 h). Improvements in physical and/or social-emotional outcomes were reported for all studies. There is emerging evidence of positive health outcomes resulting from circus activities used in general populations and those with defined biopsychosocial challenges. Future research should focus on detailed reporting of intervention elements and increasing the evidence base in preschool-aged children and within populations with the greatest need.

## 1. Introduction

The World Health Organisation’s (WHO) International Classification of Health Interventions defines a health intervention as “an act performed for, with, or on behalf of a person or a population whose purpose is to assess, improve, maintain, promote, or modify health, functioning, or health conditions” [1] (para.1). Knowledge and understanding of health interventions is essential to provide clinicians, policymakers, and service users with clarity regarding available options and the specific needs met by the interventions. Where new health interventions emerge, or activities transition to being used as a health intervention, it is essential to define the target (the body structure or function, activity, participation domain, or environmental factors being actioned by the intervention) [2], the outcomes and benefits, and the elements of the intervention itself [3]. This information may be used to inform intervention design, referrals, allocation of funding, and research [2,3]. Circus activities are one such emerging health intervention. Over the past few decades, circus activities have increasingly been used with children and young people to improve health and well-being [4], transitioning from a spectacle that people watch to an engaging recreational activity that people participate in [5]. As circus activities are increasingly being utilised to improve health and function in therapeutic [6,7], educational [8,9,10], and service settings [11], there is a clear need to define their use in this context.

The definition of participation in circus activities refers to the attendance and involvement [12] of young people in what have variously, and sometimes interchangeably, been described as social circus, community circus, youth circus, circus arts, and recreational circus [9,13,14,15]. Circus activities are typically described as having five distinct disciplines: aerials (e.g., trapeze), object manipulation (e.g., juggling), equilibristics (balancing activities such as tightwire), acrobatics (such as tumbling skills seen in gymnastics) and clowning [8,14,15]. The multi-disciplinary nature of circus presents an opportunity to focus many different activities towards a single goal or end-point, which may be particularly useful in a therapeutic context [16]. Furthermore, where children can participate in a variety of different physical activities, they may have greater opportunities for emotional regulation, identity exploration, and subsequent benefits to mental well-being [17]. Within each of the circus disciplines previously listed, there is the opportunity for a wide range of incremental challenges, meaning that participants can typically find an appropriate level of challenge, progress at their own pace and skill level, and experience early and frequent achievement leading to increased confidence and persistence [8,13,16,18,19,20,21,22].

As circus activities may present a rich and motivating health intervention opportunity, a comprehensive review of the elements of current circus interventions and their potential benefits is crucial and timely. Although a preparatory search of PROSPERO, MEDLINE, the Cochrane Database of Systematic Reviews and *JBI Evidence Synthesis* did not reveal current scoping or systematic reviews on this topic, it did reveal an emergent, heterogeneous body of literature unsuited to a more systematic review of the evidence. Therefore, the objective of this scoping review is to examine the extent and characteristics of the evidence on circus activities as a health intervention for children and young people aged up to 24 years, specifically (a) the characteristics of the participants targeted by the interventions, (b) the elements (frequency, intensity, time, type) and (c) health and wellbeing outcomes of the interventions, and (d) identification of gaps in the evidence to guide future research. This examination of the literature will allow researchers and practitioners to effectively advance the science of circus activities used as health interventions.

## 2. Materials and Methods

The review was conducted in accordance with the Johanna Briggs Institute (JBI) methodology for scoping reviews and the Preferred Reporting Items for Systematic Reviews and Meta-Analyses—Extension for Scoping Reviews (PRISMA-ScR) [23,24,25]. A protocol was developed and published by the authors a-priori to pre-define the objectives, methods, and proposed plan of the scoping review, as well as to ensure that the approach was systematic and appropriate both for the topic and the methodology [26]. A summary of the methods is provided below, with any modifications to the protocol justified and detailed.

This review is guided by the research question, “What is known about circus activities as a health intervention for children, youth, and adolescents?” Sub-questions of this review are:

- What participant characteristics (skill, developmental stage, or biopsychosocial challenges) are being targeted by the circus interventions?
- What are the key elements of the circus interventions (activities being taught; frequency, length, total number of sessions, and settings utilised)?
- What health and well-being outcomes are reported in the literature investigating circus interventions?
- What are the gaps in the current literature and how do they, and the findings of this scoping review, inform the planning of further research?

### 2.1. Eligibility Criteria

The criteria for inclusion of studies in this review have been developed based on the population, concept, and context framework related to the research questions [23]. Studies were included if participants were aged 0–24 years, or otherwise defined as children, youth, or adolescents [27,28], and the literature described or evaluated circus activities from the aerial, acrobatic, equilibristic, and manipulation categories taught to participants according to the WHO’s definition of a health intervention [1]. This review considered quantitative, qualitative, and mixed methods studies in any context to allow exploration of the variety of settings in which circus interventions are being conducted. In addition, grey literature, scoping, and systematic reviews were eligible for inclusion. No limits were placed on the publishing date, as the objective was to fully map existing literature in the area. Due to improvements in freely accessible translation services, language restrictions described in the protocol were removed, resulting in two sources of evidence [29,30] being translated into English using Google Translate (Google, Mountain View, CA, USA).

Studies were excluded if participants aged over 24 years were included, if age was not defined, or where the circus activity was not the therapeutic part (e.g., used as a warm-up only). Finally, interventions drawing solely from the “clowning” discipline of circus activities were excluded, as the evidence primarily sits in a clown doctor or clown care setting and would require a separate review.

### 2.2. Search Strategy & Study Selection

The search strategy was developed by the authors in consultation with a research librarian. A pilot search of two databases was undertaken, and additional text and index terms from identified articles were added to the search strategy (see Appendix A). To test the effectiveness of the strategy, the authors examined omitted records to ensure that no relevant studies were excluded.

Sources of information used in the final search (August 2022) were MEDLINE (Ovid: biomedical sciences), CINAHL Complete (EBSCO: nursing and allied health), Scopus (Elsevier: multidisciplinary), and PsycINFO (Ovid: behavioural science and mental health), ProQuest Dissertations and Theses Global, and Google Scholar. The strategy was adapted for each information source as required and reference lists of included sources of evidence were searched for additional eligible studies.

**Figure 1:**
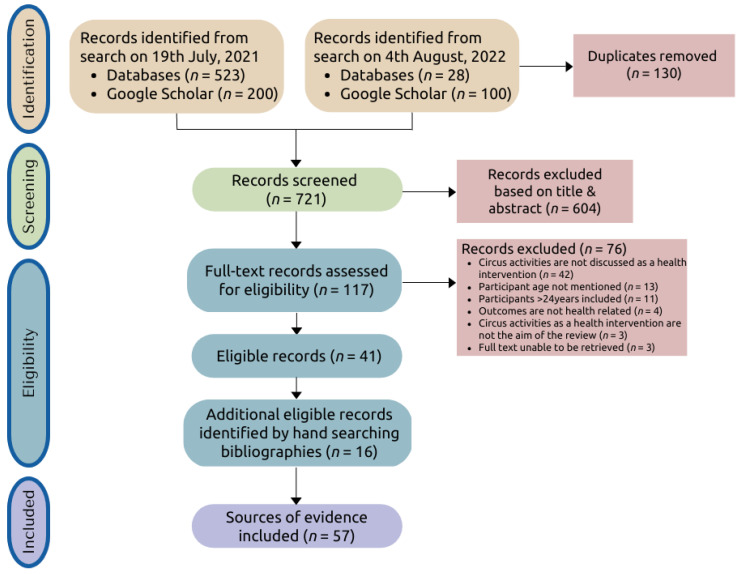
PRISMA flow diagram (adapted from [25]).

All identified records were collated and uploaded into the review management software Covidence (Veritas Health Innovation, Melbourne, Australia). Titles and abstracts were screened, and potentially relevant papers were retrieved in full and assessed in detail against the eligibility criteria. Screening was completed independently by three reviewers—F.C. screening all sources of evidence (100%), K.C. and M.C. independently screening 60% and 40%, respectively. Pilot testing to evaluate the reliability of the screening process showed 88% agreement between reviewers (75% was considered acceptable) [23]. Disagreements between reviewers were resolved through discussion with the third reviewer. Reasons for exclusion were recorded and presented in a PRISMA-ScR flow diagram (Figure 1).

### 2.3. Data Extraction and Analysis

A data extraction tool was developed a-priori (Appendix B) [26]. No modifications to the tool have been made post-protocol publication; however, due to reviewer availability, a decision was made by the review team to amend the extraction process. Data were extracted from 22 studies by two independent reviewers (F.C. and M.C) to improve rigour; however, after discussion to refine the extraction process, F.C. proceeded to extract all remaining sources of evidence (*n* = 35).

Extracted data were grouped under thematic headings relating to the review’s first three sub-questions. Data were then synthesised into a narrative summary with accompanying graphic or tabulated results. For the final sub-question, highlighted patterns, differences, and gaps in the findings across sources of evidence are explored in the Discussion, to guide future intervention development and research. Studies in a scoping review are not required to meet a quality threshold to be included, because the aim is to map and synthesise the current evidence, regardless of quality [23]. Therefore, no formal assessment of the methodological quality of the included studies was performed; however, key methodological issues affecting the validity and interpretation of findings are considered in the Discussion [31].

## 3. Results

### 3.1. Characteristics of Included Sources of Evidence

A PRISMA-ScR flow diagram (Figure 1) outlines the results of the search and screening process, including reasons for the exclusion of full-text papers [25]. An initial search conducted on 19 July 2021 resulted in a total of 723 citations, and a final search conducted on 4 August 2022 resulted in 128 citations. After de-duplication, 721 unique sources of evidence were screened at the title and abstract level, and 604 citations were excluded. The remaining 117 full texts were screened, and 41 sources of evidence were eligible for inclusion. After reviewing reference lists of included papers, another 16 sources of evidence were eligible, for the total inclusion of 57 sources of evidence.

**Figure 2:**
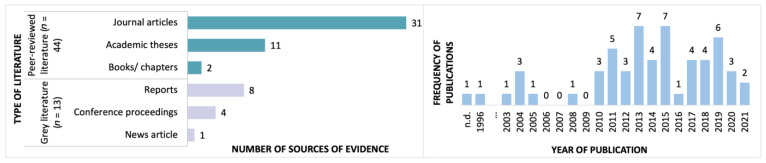
Year published and type of publication.

Within these 57 sources of evidence, a total of 42 unique interventions are described, as multiple papers described single interventions (citations are grouped by intervention in Appendix C). Most sources of evidence (46/57) were published in the past decade, with 33% (*n* = 19) published in the past five years (Figure 2).

The majority of evidence on this topic is peer-reviewed (44/57), including a book [32] and book chapter [16], academic theses [20,29,30,33,34,35,36,37,38,39,40], and journal articles [4,6,8,9,14,17,18,21,22,41,42,43,44,45,46,47,48,49,50,51,52,53,54,55,56,57,58,59,60,61,62]. No systematic or scoping reviews fulfilled the eligibility criteria. Grey literature (13/57) included project reports [63,64,65,66,67,68,69,70], conference proceedings [71,72,73,74], and a newspaper article [75].

Study designs were described in 44 of the included sources of evidence and included: randomised comparison trials (2 unique interventions) [41,42,53,54,55,56,57,60,61], case studies [30,34,35,37,51,59,62], qualitative studies [14,21,22,29,38,40,45,46,68,73,74], mixed-methods [4,47,63,64,65,67], pre- post- designs [6,66], a cross-sectional design [17], a between-subjects repeated measures design [50], and a prospective, clustered quasi-experimental design (one unique intervention) [8,36]. Sample sizes ranged between one and 300 participants, and further details can be found in Appendix D.

### 3.2. Characteristics of Participants Targeted by Circus Interventions

This scoping review included participants aged up to 24 years, or otherwise defined as children, youth, or adolescents to allow examination of the developmental stage (e.g., pre-school), biopsychosocial challenge (e.g., cerebral palsy, mental illness, or homelessness) and/or skill (e.g., motor or social), being targeted by the circus interventions.

The development stages of participants in included studies may be grouped into pre-school (*n* = 3) [58,68,69], school-aged (*n* = 43) [6,8,9,14,16,18,20,22,30,32,33,34,36,37,38,39,41,42,44,45,47,48,49,50,51,52,53,54,55,56,57,60,61,62,63,66,67,69,70,73,74,75], and older adolescents and youth (*n* = 5) [4,17,30,35,46,64]. Age ranges larger than 15 years were present in four papers, with participants ranging from pre-school to post-school age [21,65,71,73]; six sources of evidence did not specify the age of the participants but described the inclusion of “children” [40,72], “youth” [43,64], or both [29,59]. Further details and mean ages (where they could be calculated) are presented in Appendix D.

Twenty of the included sources of evidence included participants from general populations. The remaining papers (37/57) included participants with defined biopsychosocial challenges such as cerebral palsy [41,42,45,46,48,53,54,55,56,57,60,61], vision impairment [69], a range of intellectual and/or physical disabilities [29,46,62], moderate to severe learning difficulties [65], overweight [49], developmental difficulties without a specified medical condition or disability [22], neurodiverse children and youth (including autism) [6,40,58,64,69], youth in residential psychiatric care [4,30,44,66,73], children and youth living in or exposed to warzones [21,35,72], and those described as “living on the street” [59], “at risk” [29,37,63,66], “vulnerable” [51,69], or as having “special needs” [69].

### 3.3. Key Elements of the Circus Interventions

To describe the circus activities being utilized as a health intervention (i.e., the “concept” of the scoping review [23]), the activities conducted in each intervention (the “What” [3]), the setting of the interventions (the “Where” [3]), and the dosage (“When and how much” [3]) are presented in detail in Appendix C. Only 15 of the 42 unique interventions fully describe each of these elements. The specific circus activities (the “What” [3]) can be categorised according to the five circus disciplines [26]. As can be seen in Table 1, 26 of the 38 interventions that described the circus activities conducted, draw from three or more circus disciplines, and five interventions draw from all five of the disciplines.

**Figure 3:**
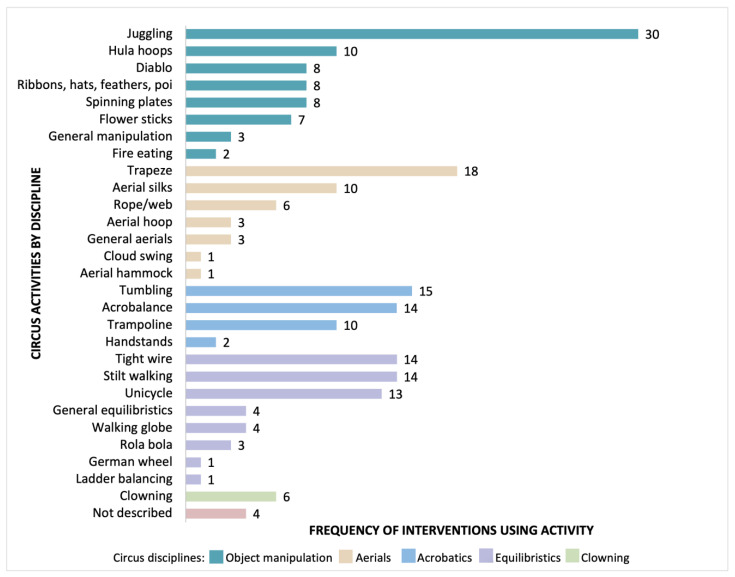
Circus activities utilised in interventions.

Figure 3 describes how often the individual circus activities were utilised in the interventions. Juggling and trapeze were utilised most often (in *n* = 30 and *n* = 18 interventions respectively). When looking at each circus discipline, object manipulation activities (which include juggling) were utilised most often in interventions (76 instances), equilibristic (balancing) activities were utilised in 54 instances, both aerial activities in 42 instances, acrobatic activities in 41 instances, and clowning in six interventions.

**Figure 4:**
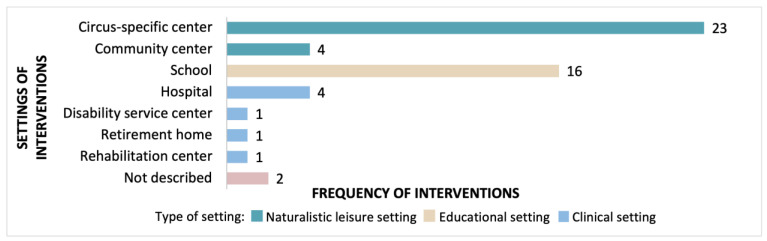
Settings utilised in circus interventions.

Figure 4 describes the “Where” [3] or context [23] of the interventions. More than half (27/42) of the unique interventions utilised naturalistic leisure settings, such as circus-specific centres [6,9,14,16,17,20,22,29,32,34,35,38,40,41,42,43,45,46,47,48,49,52,53,54,55,56,57,58,60,64,68,69,70,71,72] or community centres [4,21,51,72]. Educational settings (primary and secondary schools) were utilised in 16 unique interventions [8,9,17,18,33,36,37,39,50,62,63,65,66,67,69,72,73,74,75], and five sources of evidence describe interventions delivered in seven medical settings [30,44,66,69,73]. Eight sources of evidence reported multiple settings for the circus interventions [9,17,40,58,66,69,72,73]; three sources of evidence (two unique interventions) did not report the setting [43,59,70].

Table 1 illustrates the dosage (“When and how much” [3]) of the circus interventions. Circus sessions were most commonly delivered once (*n* = 19) or twice (*n* = 10) per week. Two interventions ran in an intensive model with five circus sessions per week for one [72], and two weeks respectively [41,42,45,48,53,54,55,56]. The duration of the circus activities ranged from 45-min to five hours, with a mode of one hour. Total intervention dosage ranged from 1–96 h in 15 interventions but could not be calculated in the remaining interventions due to a lack of detailed reporting. Sixteen studies (17 sources of evidence) investigated the effects of ongoing programs rather than a defined, separate intervention [14,20,29,30,35,37,38,40,43,44,51,52,62,68,69,70,71].

### 3.4. Outcomes Relating to Health & Wellbeing

Overall, the timing of outcome measurement was variable. For 24 interventions (31 sources of evidence), outcomes were assessed immediately following the intervention [8,9,22,34,36,47,49,50,53,54,55,56,57,60,61,63,64,65,66,67,70] or during the intervention [21,45,48], with three studies conducting additional mid-point data collection [6,46,70]. Five studies (nine sources of evidence) conducted additional three-month follow-up assessments [4,39,53,54,55,56,57,60,61], and one study (two sources of evidence) conducted 52-week follow-up assessments [60,61]. For participants in ongoing programs (12 sources of evidence), data was collected at a single timepoint in most cases [14,17,20,35,37,51,52,68,69,71]; however, one qualitative study collected data over a 12-month period [29], and yet another conducted a follow-up with participants four and five years after the initial data collection [38]. One study utilising outcome measures did not report the timepoint of data collection [59], and eight sources of evidence provided observations of circus activities used therapeutically with no assessment timepoints described [30,33,40,44,58,62,72,73,75].

**Figure 5:**
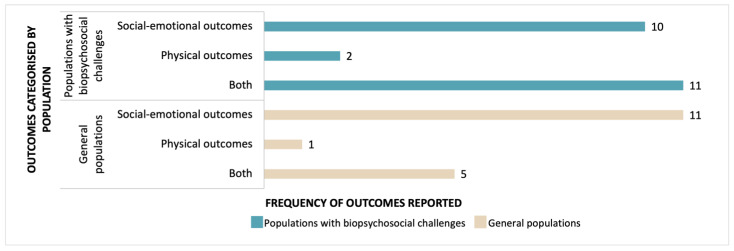
Categorisation of outcomes of circus interventions.

The outcomes aiming to enhance health and well-being varied depending on the characteristics of the participants (as described earlier in this paper) but can be categorised into physical and/or social-emotional outcomes (see Figure 5 and Appendix E for details relating to extracted outcomes). Physical outcomes were targeted for children with cerebral palsy [41,42,45,48,53,54,55,56], those classified as overweight [49], as well as general populations [52]. Social-emotional outcomes were targeted in children exposed to warzones [21,35,72], those considered “at risk” [37,66], or “living on the street” [59], young people in residential psychiatric care [30,73] or with a range of intellectual [62] and/or physical disabilities [46], and those in general populations [9,14,17,33,34,39,43,47,67,70,71,75].

Both physical *and* social-emotional outcomes were targeted for children with vision impairment or “special needs” [69], children described as “vulnerable” [51], “at risk” [29,63] neurodiverse or children with autism [6,40,58,64], those with vision-impairment [69], with developmental difficulties [22], learning difficulties [65], in residential psychiatric care [4], as well as in general populations [8,18,20,36,38,50,68,74].

### 3.5. Physical Outcomes

Physical outcomes of circus interventions reported included increased participation in physical activity [8,18,36,50,74] or decreased inactivity [4], improved motor competence [8,18,36,69,74] or “physicality” [6], increased postural control [52], improved bimanual and occupational performance and unimanual upper-limb capacity [45,54,55,56,57], reduced lymphocyte proliferation, improved immune system function [49], as well as increases in fitness, strength, flexibility, coordination, body awareness [20,22,29,38,40,51,58,64,69], and acquisition of new physical skills [29,63,64,65,68].

### 3.6. Social-Emotional Outcomes

Social-emotional outcomes reported included building resilience and perseverance [14,21,36,39,48,60,64,70], developing confidence and/or self-esteem [8,9,14,18,20,21,22,30,35,36,40,46,47,51,62,63,64,65,66,67,68,69,74], enhancing development of social skills and connection [6,9,14,21,21,22,29,38,40,46,47,58,59,64,65,66,67,68,69,73,75], and fostering a sense of inclusion and belonging [21,29,35,58,59,67,68] including increased community participation [46]. Circus activities also resulted in increased intrinsic motivation, positive affect and grit [17], and were used to successfully build relationships in communities [33]. Positive impacts on mental well-being were reported [14,47], as was the growth of social-emotional skill development such as emotion management, teamwork, initiative, empathy, responsibility, and problem-solving [30,43,70]. Circus interventions resulted in improved communication [29,34,46,62,63,65,72] and emotional regulation skills [6,29,30,34], enhanced happiness or positive affect [20,21,69,71,73], and a decrease in destructive or “difficult” behaviours [37,68,72].

One study reported a decrease in the “emotional symptoms” subscale of the Strengths and Difficulties Questionnaire but no other differences were detected [50]. There were also no differences in psychosocial functioning for children participating in a circus program compared with those who were not [39], and in a qualitative study with young people in psychiatric care, participants reported no difference in their social skills, relationships, or self-esteem; however, the participants’ physicians reported substantial differences in the same constructs [4]. Another qualitative study reported improvements in children’s social-emotional development in circus class but noted that these improvements did not appear to cross over to schooling or home life [29].

The use of circus activities also increased motivation to participate in therapy [44,45] and physical activity [9,14,21,69,73] and improved quality of life for participants [60]. A Social Return on Investment analysis found “for every dollar invested, AUD$7 of social return may be generated due to participation in a circus program” [47] (p. 163).

## 4. Discussion

Circus activities are emerging as an engaging and unique health intervention [8]. Calls to define the use of circus activities in therapeutic contexts have come from circus practitioners themselves and more generally, as part of a movement toward clear, accessible intervention descriptions to inform future intervention design, referrals, allocation of funding, and research [2,3,6,30]. The current scoping review has identified an emerging evidence base of peer-reviewed and grey literature regarding the use of circus activities as a health intervention for children and young people. The final aim of this scoping review is to explore the implications and limitations of the synthesised evidence to inform the planning of further research.

Circuses’ potential for progressive challenge facilitates individual goal setting that aligns well with contemporary motor learning approaches, as does the focus on repetitive task practice within circus activities [16,20,76]. This person-centred approach means that circus activities can be modified to suit the child rather than requiring the child to change to suit the class [13,40]. Another person-centred aspect of circus activities is the appreciation of creativity, inventiveness and out-of-the-box approaches to skill accomplishment [20,77,78]. Encouraging self-expression and individual performance of the skill (as opposed to the *right* way to complete the skill) allows participants to accomplish tasks in ways that work for them and their bodies [18,35,77]. Participation in creative activities can have a positive effect on behaviour, self-confidence, self-esteem, relationship building, sense of belonging, and mental well-being [79,80,81]. Furthermore, the compatibility and collaboration of circus with other creative art forms, e.g., dance, theatre, music, fine arts adds to its appeal, as the hybrid nature of circus can provide “something for everyone” [14,15,18,19,20,35], [69] (p. 12).

Circus activities are also highly motivating due to their potential to satisfy the psychological needs of autonomy, competence, and relatedness [8]. Respecting participant preference and choice within the variety that circus activities offer may foster autonomy [16,17,42,48], and collaboration and cooperation with peers (as opposed to competition) to work toward shared goals in circus activities can support relatedness [16,17,48]. Lastly, circus activities can enhance participants’ sense of competence through the multitude of incremental challenges available within the multi-disciplinary offerings (e.g., aerials, object manipulation, equilibristics etc.). With appropriate scaffolding of activities, participants can find an appropriate level of challenge and experience early success [8,13,16,18,20,42,45]. When these psychological needs are satisfied, participants are more motivated and experience enhanced well-being, thus circus activities are an inherently satisfying and highly motivating intervention promoting participation, internal regulation, and persistence [48,82]. The unique community-based circus-specific centres that were the primary delivery sites in this review may also contribute to the motivating nature of circus activities, as research has demonstrated that children are more likely to participate longer and be more engaged when interventions are delivered in authentic, fun and novel settings [8,16,17,45,48,83]. All of these features align well within a biopsychosocial model of health, such as the WHO’s International Classification of Functioning, Disability and Health: children and youth version (ICF-CY) [16,84].

The ICF-CY framework draws connections between children’s participation, environment, personal factors, body structure, function, and activity, and their influence on health and development [84]. Rosenbaum and colleagues (2011) have further developed the ICF to provide a specific focus on six key areas (the F-words) of child development [85]. Interventions that can target multiple F-words have the potential to impact children’s health and development across multiple domains, and the findings of this review show promise for circus activities to enhance multiple physical and social-emotional outcomes in heterogeneous paediatric populations. Circus activities can be *fun* [45,46,68,77], they can increase physical and social-emotional *fitness* [20,22,29,30,40,69], and can be undertaken with peers (*friends*), providing opportunities for social connections [6,9,14,40,47]. Circus activities are often community-based and inclusive (*future*), and typically have a strong focus on person-centred *functioning*, that is, the individual performance of skills in a non-competitive environment [8,35]. They also have the potential to be *family*-centred, as a community-based recreational activity, and where a child and family’s goals can be incorporated into a fun recreational activity, positive therapy experiences have been reported [16].

Whilst circus interventions appear to have a role in all populations studied, increasing the evidence in populations with defined biopsychosocial challenges is recommended, as directing resources towards those most in need promotes equitable health outcomes [86]. Cerebral palsy was the most explored condition, with 15 sources of evidence (26%) describing circus activities conducted with this population. The majority of this evidence looked at young people aged 5–16 years with unilateratal hemiplegia who performed an intensive circus camp for 60 h, including 20 h of activities drawn from the aerial, acrobatics and object manipulation circus disciplines. Outcomes described in these sources of evidence align with the broader literature in this scoping review, with improvements described in both physical (bimanual and occupational performance, unimanual upper limb capacity) and social-emotional domains (engagement, motivation, confidence, social connection, and communication). For children with defined biopsychosocial challenges such as cerebral palsy, the provision of interventions early in life that show impact in physical and social-emotional domains aligns with literature on early intervention [87,88]. The potential impact of interventions is greatest in early childhood where rapid development and neural plasticity can be optimised to minimise neurodevelopmental impairments [87]. For example, long-term benefits such as improvements in academic outcomes, increased rates of employment and higher incomes have been demonstrated with early intervention in the preschool age group [88]. In the current review, only three studies looked at pre-school-aged children specifically, and only one of these looked at children with a defined biopsychosocial challenge. The scarcity of studies in early childhood, as well as the demonstrated benefits of early intervention may indicate a need for further studies in this population. Furthermore, although participant and intervention characteristics should vary depending on the aim of the study, individual and family-based goals are often related to the developmental stage and/or biopsychosocial challenges of the children [16]. Therefore, in future studies utilising participants across a >15-year range, or simply describing them as “children” or “youth” without an age specified, sampling should be strongly justified.

The positive results reported in all populations studied may indicate the potential generalisability of circus interventions; however, only seven studies involved long-term follow-up of outcomes. The inclusion of long-term follow-up is recommended in future research to determine retention of the positive results reported. Furthermore, future studies should determine the optimal intervention characteristics to improve these outcomes. With just over one third of the interventions providing a full description of the elements of the circus interventions, the use of the Template for Intervention Description and Replication (TIDieR) is recommended in future research to improve the reporting and replicability of circus interventions [3]. This encourages researchers to be clear about the essential elements of the interventions, thereby providing clarity for practitioners and service users. In those studies that did report the intervention content, most interventions drew from three or more circus disciplines, in most cases object manipulation, equilibristics, and aerial apparatus. This indicates that interventions contain activities that promote manual dexterity and hand-eye coordination, balance, and upper limb and core strength, respectively. There is evidence to suggest that the most common activity present in interventions, juggling, may improve neuroplasticity and reduce anxiety in adults [89,90]. Continuing to embrace the natural variety of circus activities in future research will ensure that “everyone is good at something” [69] (p. 18) and that participants can work toward individual goals while benefitting from social interaction with peers [8].

Regarding the limitations of the present review, scoping methodology does not seek to assess the quality of evidence and consequently cannot determine whether studies provide robust or generalisable findings or assess the “weight” of the evidence concerning particular studies [23]. Papers excluded in this review for having participants over the age of 24 years often also contained participants aged 18–24 years, which could account for the few studies included with participants in the 19–24 age group.

## 5. Conclusions

The findings of this review show promise for circus activities as a health intervention for children, youth and adolescents. This review found that the characteristics of participants described in the included sources of evidence were primarily school-aged, with fewer studies investigating participants from pre-school (0–5 years) and school-leaver (18–24 years) populations. Participants were drawn from both general populations and those with defined biopsychosocial challenges, and cerebral palsy was the most commonly described condition in the included literature.

The interventions described were most often delivered in authentic community-based settings and included activities from three or more of the circus disciplines; however, detailed reporting of intervention content was lacking. Outcomes of these interventions included improvements in both physical and social-emotional domains in general populations and those with defined biopsychosocial challenges; however, most studies did not include long-term follow-up.

Future research should focus on populations where the potential impact is greatest, such as pre-school-aged children with defined biopsychosocial challenges, and ensure long-term follow-up to understand retention of outcomes. Continuing to harness the unique motivating nature of circus activities, including variety, progressive challenge, and community-based settings is recommended. However, clear reporting of interventions using a framework such as the TIDieR checklist is essential to understand the crucial elements of circus interventions and investigate their impact on physical and social-emotional outcomes. These recommendations will enable practitioners and researchers to understand, replicate, and advance these approaches, and provide clarity for clinicians and service users regarding the use and impact of circus programs. Due to a lack of homogeneity in populations and study designs, a systematic review is not recommended at this time.

## Figures and Tables

**Table 1:** Characteristics of the circus interventions.

	*Intervention Elements*				*Circus Disciplines*				
Citations Grouped by Intervention	*Sessions per Week* *(Number)*	*Total* *Sessions (Number)*	*Duration per* *Session (Hours)*	*Total* *Dosage (Hours)*	*Aerials*	*Acrobatics*	*Object* *Manipulation*	*Equilibristics*	*Clowning*
**Agans et al., 2019** [17]	Not described				X ^1^	X	X	X	X
**Barnett et al., 2020** [18]**; Kiez, 2015** [36]**; Kriellaars et al., 2019** [8]**; Valentini et al., 2020** [74]	1–4	10–84	1	10–84	X	X	X	X	
**Biquet, 2014** [35]	1	ongoing program	3		X	X	X	X	
**Bolton, 2004** [33]			2		-	X	X	X	
**Bonk, 2019** [34]	1	1	1	1	X	X	X	X	
**Boyd et al., 2010** [42]**; Gilmore et al., 2010** [45]**; Rodger & Kennedy-Behr, 2017** [32]**; Sakzewski et al., 2012** [60]**; Sakzewski, Provan, et al., 2015** [57]**; Sakzewski, Ziviani, Abbott, et al., 2011a** [54]**, 2011b** [55]**, 2011c** [61]**; Sakzewski, Ziviani, & Boyd, 2011** [53]**; Sakzewski, Ziviani & Poulsen, 2015** [16]**;**	5	10	2 (in addition to 4 h of other activities)	20 (of circus activities)	X	X	X		
**Boyd et al., 2013** [41]**; Miller et al., 2016** [48]**; Sakzewski, Miller, et al., 2015** [56]	5	10	2	20	X	X	X		
**Cadwell & Rooney, 2013** [71]		ongoing program			Specific circus activities not described				
**Caldwell, 1996** [75]	Not described						X		
**Candy, 2017** [20]	1 or 2	ongoing program	2.5		X		X	X	
**Cohen, 2018** [43]**; Smith et al., 2017** [70]		ongoing programs			Specific circus activities not described				
**Csuros, 2015** [44]	1	ongoing program				X	X	X	
**Fernandez et al., 2018** [22]	1	10	1	10	X	X			
**Fournier et al., 2014** [4]	2	24			Specific circus activities not described				
**Heller & Taglialatela, 2018** [6]	1	16	1	16	X		X	X	
**Kinnunen et al., 2013** [69]		ongoing program			X	X	X	X	
**Kovalenko, 2018** [29]	5	ongoing program	1.5–3			X	X		
**Loiselle et al., 2019** [46]	2	48	2	96	X	X	X	X	X
**Maglio & McKinstry, 2008** [9]	1	10–40	1	10–40		X	X	X	
**Mason, 2013** [72]	5	5				X	X		
**McCaffery, 2011** [65]	1	18	1.5–2	27–36			X	X	
**McCaffery, 2012** [64]		18	1.5	27			X	X	
**McCaffery, 2014** [63]	1	8	1.5	12			X	X	
**McCutcheon, 2003** [37]		ongoing programs			X	X	X	X	X
**McGrath & Stevens, 2019** [47]	1	20	1	20	X	X		X	
**Momesso dos Santos et al., 2015** [49]	2	38	1	38	X	X		X	
**Neave et al., 2020** [50]	1	18	1	18	X	X	X	X	
**O’Donnell, n.d.** [66]	1		1			X	X	X	X
**Ott, 2005** [38]	2	ongoing program	2		X	X	X	X	X
**Pompe, 2021** [30]		ongoing program				X	X		
**Rappaport, 2014** [73]	1–2		0.75–1.25		X	X	X	X	X
**Rivard et al., 2010** [51]	1–2	ongoing program				X	X		
**Sahli et al., 2013** [52]	2	ongoing program	2				X	X	
**Seay, 2004** [39]	Not described				X	X	X		
**Seymour, 2012** [40]	1	ongoing program			X	X	X	X	
**Seymour & Wise, 2017** [58]	1				X	X	X		
**Spiegel et al., 2015** [59]	Not described				Specific circus activities not described				
**Stevens et al., 2019** [14]		ongoing program			X	X		X	
**Taylor & Taylor, 2004** [62]	3	ongoing program	1				X	X	
**Trotman, 2013a** [67]	1	8	1.5	12		X	X	X	
**Trotman, 2013b** [68]	1	ongoing program	1				X	X	
**Van Es et al., 2021** [21]	2	16	5	80	X	X	X	X	

^1^ X denotes the circus activity was described as part of the intervention.

## Data Availability

The data presented in this study are available in the reference list.

## Appendix A. Search Strategy Development

**Table A1:** Pilot search strategy for MEDLINE (Ovid) and CINAHL.

	Population 1	Concept 1	Concept 2
As stated in research question	Children and youth	Circus activities	Health intervention
Alternate search terms for those concepts, using truncation * and wild cards # or?	paediatr*, preschool, kinder, kids, adolescent, infant, school-aged, young people	Social circus, community circus, recreational circus, youth circus, hula hoop, juggl*, acroba*, trapeze, tissu, silks	therap*, physiotherapy, occupational therapy, psychology*, program, exercise*, activit*, class, classes
Synonyms (alternate words) alternative spelling, language, etc.	Pre-school		Programmes, programme, programs
Discarded after trialing in MEDLINE and CINAHL, with reasons	All population terms discarded due to scarcity of literature.	acroba*: no items returned relating to circus, many returned relating to gymnastics juggl*: first 50 citations screened related to “juggling work/life”, and none to circus trapeze: first 50 citations screened did not relate to circus tissu OR silks: first 50 citations screened did not relate to circus	
Indexed key words added after pilot search in MEDLINE and CINAHL	Nil	Nil	“social work”

**Table A2:** Example of search strategy in MEDLINE (Ovid).

Search	Query	Records Retrieved
#1	circus OR hula hoop* OR hula-hoop*	699
#2	therap* OR intervention* OR class OR classes OR program OR programme OR programs OR programmes OR activit* OR exercise OR exercises OR physiotherap* OR social work* OR psycholog*	11,234,666
#3	#1 AND #2	276
Limited to ‘human’		165

## Appendix B. Data Extraction Instrument

**Table A3:** Data extraction table used in Scoping Review.

Element for Extraction	Extracted Data
Citation: author/s, date	
Country of intervention delivery	
Literature type; Study design	
Sample size	
Participant details: (Aim 1)	
age range and mean	
characteristic targeted by intervention	
Intervention details (adapted from TIDieR checklist [3]): (Aim 2)	
total duration of intervention	
number of sessions per week	
total number of sessions	
length of sessions	
total dosage (number of sessions x length of sessions)	
circus activities described	
Health/ wellbeing outcomes relating to circus intervention (Aim 3)	

## Appendix C. Elements of the Circus Interventions

**Table A4:** The “what”, “where”, “when”, “how much” and “for whom” of interventions [3].

Citations Grouped by Intervention	What (Circus Activities Only)	Where	When & How Much	For Whom
Agans et al., 2019 [17]	Tumbling, juggling, clowning, tight wire, trapeze	School, circus centre	Ongoing program, dosage not described	General population (circus school)
Barnett et al., 2020 [18]; Kiez, 2015 [36]; Kriellaars et al., 2019 [8]; Valentini et al., 2020 [74]	Juggling (balls, scarves), rola bola, stilt walking, unicycle, trampoline, flower sticks, trapeze, aerial rope, hula hoops, tight wire, German wheel, diabolo	School	One to three 50–60 min sessions per week for a school year	General population (primary school)
Biquet, 2014 [35]	Juggling, acrobalance (human pyramids), tumbling, general aerial skills, stilt walking	Circus centre	Once per week for 3 h in an ongoing program	“living in a warzone”
Bolton, 2004 [33]	Juggling, stilt walking, unicycle, acrobalance	School	2 h per session for two weeks	General population (ex-pats & local community)
Bonk, 2019 [34]	Handstands, tumbling, juggling, unicycle, aerial silks, aerial hoop, and trapeze	Circus centre	One 1-h session	General population (circus school)
Boyd et al., 2010 [42]; Gilmore et al., 2010 [45]; Rodger & Kennedy-Behr, 2017 [32]; Sakzewski et al., 2012 [60]; Sakzewski, Provan, et al., 2015 [57]; Sakzewski, Ziviani, Abbott, et al., 2011a [54], 2011b [55], 2011c [61]; Sakzewski, Ziviani, & Boyd, 2011 [53]; Sakzewski, Ziviani & Poulsen, 2015 [16]	Ribbons, flower sticks, hula hoops, spinning plates, acrobalance, general aerials	Circus centre	Five 2-h sessions (in combination with 4 h of other tasks) for 2 weeks	Cerebral palsy: unilateral hemiplegia
Boyd et al., 2013 [41]; Miller et al., 2016 [48]; Sakzewski, Miller, et al., 2015 [56]	Ribbons, flower sticks, hula hoops, spinning plates, acrobalance, general aerials	Circus centre	Five 2-h sessions (within 4 h of other tasks) for 2 weeks	Cerebral palsy: unilateral hemiplegia
Cadwell & Rooney, 2013 [71]	Specific circus activities not described	Circus centre	Ongoing program, dosage not described	General population (circus school)
Caldwell, 1996 [75]	Juggling	School	Ongoing program, dosage not described	General population (circus school)
Candy, 2017 [20]	Juggling (scarves, balls, clubs, rings), balance feathers, spinning plates, diablo, unicycle, tightwire, hula hoops, rola bola, stilt walking, aerial silks, trapeze, rope	Circus centre	One or two 2.5 h sessions per week in an ongoing program	General population
Cohen, 2018 [43]; Smith et al., 2017 [70]	Specific circus activities not described	Not described	Between 30 and 489 contact hours depending on the program	General population (circus school)
Csuros, 2015 [44]	Juggling, tumbling and tightwire	Hospital	One session per week in an ongoing program	Children in psychiatric care: “conduct disorder, hyperactivity, attention deficit disorder (ADD), autism, spectrum disorders and psychiatric disorders requiring emergency care (crisis conditions, suicidal intensions or attempted suicide, self-destructive behaviour, sudden psychotic conditions), schizophrenia, mood disorders, anxiety disorders, post-traumatic disorder, emerging personality disorders, obsessive compulsive disorder, eating disorders, attachment disorders.”
Fernandez et al., 2018 [22]	Trapeze, aerial hoop, trampoline, tumbling	Circus centre	One 60 min session per week for 10 weeks	Children with developmental difficulties (without a specified medical condition or disability).
Fournier et al., 2014 [4]	Specific circus activities not described	Community centre (YWCA)	Two sessions per week for 3 months	Children in psychiatric care: mood disorders, anxiety disorders, relational or behavioural problems, attention deficit hyperactivity disorder (ADHD), psychotic disorders, eating disorders.
Heller & Taglialatela, 2018 [6]	Rola bola, hula hoops, aerial hammocks, trapeze, aerial web, aerial ring, tightwire	Circus centre	One 50–60 min session per week for 2 eight-week blocks	ADD, ADHD, anxiety, autism, behavioural challenges, impulse control challenges (sourced from personal communication with author).
Kinnunen et al., 2013 [69]	Hula hoops, aerial silks, trampoline, trapeze, unicycle, juggling, tight wire, acrobalance, diablo, handstands	School, rehabilitation centre, circus centre, retirement home, disability service centre	Varying dosages for different settings	Variety of populations, including “special needs” class (school); general population (school & pre-school); ADD/ ADHD, autism; children of families who use the child welfare services and are dealing with crises; children with visual impairment.
Kovalenko, 2018 [29]	Trampoline, juggling	Circus centre	Five 1.5–3-h sessions per week in an ongoing program	“at risk”: children from difficult family circumstances, adolescents who have committed an offence, children with disability.
Loiselle et al., 2019 [46]	Tumbling, trapeze, aerial silk, juggling, clowning, equilibristics	Circus centre	Two 2-h sessions per week for two 12-week blocks	Young people with physical disabilities including: cerebral palsy, leukodystrophy, Steiner’s syndrome, Childhood callosotomy sequelae for uncontrolled epilepsy, Friedrich’s ataxia, traumatic acquired brain injury (ABI), severe burns in infancy
Maglio & McKinstry, 2008 [9]	Tumbling, acrobalance, juggling, hula hoops, equilibristics	Circus centre, school	One 2-h session per week for 10–40 weeks	General population (school)
Mason, 2013 [72]	Juggling, tumbling	Circus centre, school, community centre	One session per day for 5 days	“living in a warzone”
McCaffery, 2011 [65]	Juggling, unicycle, stilt walking, flower sticks, poi, diabolo, spinning plates, hat manipulation	School	One 90–120 min session per week for 18 sessions	School students with moderate to severe learning difficulties
McCaffery, 2012 [64]	Juggling, unicycle, stilt walking, flower sticks, poi, diabolo, spinning plates, hat manipulation	Circus centre	One 90 min session per week for 18 sessions	Autism
McCaffery, 2014 [63]	Juggling, stilt walking, unicycle, general manipulation, general equilibristics	School	One 90 min session per week for 8 weeks	“at risk” school students: at-risk of disengagement, poor attendance or leaving school early.
McCutcheon, 2003 [37]	Unicycle, aerial silks, acrobalance, juggling, diabolo, stilt walking, tumbling, flower sticks, rola bola, clowning, ladder, trapeze, cloud swing, tightwire, trampoline	School	Varying dosages for different students	“at risk” school students
McGrath & Stevens, 2019 [47]	Stilt walking, trampoline, tumbling, trapeze, aerial silks	Circus centre	One 60 min session per week for 20 weeks	General population (circus school)
Momesso dos Santos et al., 2015 [49]	Tumbling, trampoline, stilt walking, trapeze	Circus centre	Two 60 min session per week for 38 sessions	Overweight
Neave et al., 2020 [50]	Acrobalance, aerial rope, unicycle, juggling	School	One 60 min session per week for 6 months	General population (school)
O’Donnell, n.d [66]	Stilt walking, tight wire, walking globe, tumbling, juggling, clowning, general manipulation	Schools, hospital	One 60 min session per week for ‘several weeks’	“at-risk” due to socioeconomic status, single-parent homes, and/or a history of truancy and disruptive classroom behaviour at school; inpatient psychiatric unit.
Ott, 2005 [38]	Trapeze, tumbling, juggling, clowning, aerial web, tight wire, unicycle, fire-eating	Circus centre	Two 120 min session per week in an ongoing program	General population (circus school)
Pompe, 2021 [30]	Acrobalance, juggling, trampoline	Hospital	Ongoing program	Inpatient psychiatric unit
Rappaport, 2014 [73]	Acrobalance, juggling, tight wire, trapeze, aerial silks, clowning	School, hospital	(a) One 75 min session per week (b) Two 45 min sessions per week	Inpatient psychiatric unit: mood disorder, bipolar, depression; deaf, hard of hearing, or other communication difficulties.
Rivard et al., 2010 [51]	Acrobalance, juggling	Community centre	Once or twice per week in an ongoing program	Vulnerable young people in “difficult situations”
Sahli et al., 2013 [52]	Stilt walking, rola bola, walking globe, juggling and other manipulation, unicycle, hula hoops, tight wire	Circus centre	Two 2-h sessions per week in an ongoing program	General population (circus school)
Seay, 2004 [39]	Juggling, aerial silks, fire eating, trampoline, tumbling, trapeze	School	Two-week program	General population (school)
Seymour, 2012 [40]	Trapeze, juggling, tight wire, hula hoops, acrobalance	Circus centre, other various	Not described	Autism and other neurodiversities
Seymour & Wise, 2017 [58]	Trapeze, acrobalance, juggling, aerial silks	Circus centre, other various	Not described	Autism
Spiegel et al., 2015 [59]	Specific circus activities not described	Not described	Not described	Youth “living on the street”
Stevens et al., 2019 [14]	Stilt walking, trampoline, tumbling, trapeze, aerial silks	Circus centre		General population (circus school)
Taylor & Taylor, 2004 [62]	Juggling (scarves, bean bags, balls), flower sticks, spinning plates, diabolo, poi, stilt walking, unicycle	School	Three 60 min sessions per week in an ongoing program	Range of intellectual and/or physical disabilities (mild to severe)
Trotman, 2013a [67]	Spinning plates, diabolo, tight wire, walking globe, hula hoops, tumbling, acrobalance	School	One 90 min session per week for 8 weeks	General population (school)
Trotman, 2013b [68]	Juggling, equilibristics	Circus centre	One 60 min session per week in an ongoing program	General population (circus school)
Van Es et al., 2021 [21]	Tumbling, trapeze, juggling, spinning plates, tight wire, walking globe	Community centre	Two 5-h session per week for 8 weeks	Refugee youth/ children and youth with traumatic war experiences.

## Appendix D. Sources of Evidence Included

**Table A5:** Type and design of evidence and participant details.

Citations	Type of Literature	Study Design	Sample Size	Age Range (Mean)
Agans et al., 2019 [17]	Peer-reviewed: journal article	Cross-sectional design	111	10–21 y (15 y 9 m)
Barnett et al., 2020 [18]	Peer-reviewed: journal article	Concept paper	N/A	9–12 y (10 y 0 m)
Biquet, 2014 [35]	Peer-reviewed: masters thesis	Case study	15	11–20 y
Bolton, 2004 [33]	Peer-reviewed: PhD thesis	Not described	30	Kindergarten-Year 10
Bonk, 2019 [34]	Peer-reviewed: masters thesis	Case study	1	10 y
Boyd et al., 2010 [42]	Peer-reviewed: journal article	Randomised comparison trial protocol	N/A	N/A
Boyd et al., 2013 [41]	Peer-reviewed: journal article	Randomised comparison trial protocol	N/A	N/A
Cadwell & Rooney, 2013 [71]	Grey literature: conference proceedings	Not described	33	5–20 y
Caldwell, 1996 [75]	Grey literature: newspaper article	N/A	Not described	6–11 y
Candy, 2017 [20]	Peer-reviewed: masters thesis	N/A	11 children 18 parents	“school-aged children”
Cohen, 2018 [43]	Peer-reviewed: journal article	Not described	Not described	“youth”
Csuros, 2015 [44]	Peer-reviewed: journal article	Not described	10–15	4–18 y
Fernandez et al., 2018 [22]	Peer-reviewed: journal article	Qualitative	20	5–10 y
Fournier et al., 2014 [4]	Peer-reviewed: journal article	Mixed-methods	15 patients 9 non-patients	14–24 y
Gilmore et al., 2010 [45]	Peer-reviewed: journal article	Qualitative	32	9–11 y (10 y 1 m)
Heller & Taglialatela, 2018 [6]	Peer-reviewed: journal article	Pre-post survey design	15	4–12 y (6 y 6 m)
Kiez, 2015 [36]	Peer-reviewed: masters thesis	Prospective, clustered, quasi-experimental design	211	9–12 y (10 y 0 m)
Kinnunen et al., 2013 [69]	Grey literature: report	Mixed-methods	Not described	2–3 y, “babies”, “primary and high-school aged children”, 7–13 y
Kriellaars et al., 2019 [8]	Peer-reviewed: journal article	Prospective, clustered, quasi-experimental design	211	9–12 y (10 y 0 m)
Kovalenko, 2018 [29]	Peer-reviewed: bachelors thesis	Qualitative	7	“children & adolescents”
Loiselle et al., 2019 [46]	Peer-reviewed: journal article	Qualitative	11	18–22 y (20 y 0 m)
Maglio & McKinstry, 2008 [9]	Peer-reviewed: journal article	Process evaluation	Not described	6–18 y
Mason, 2013 [72]	Grey literature: conference proceedings	Not described	Authors have educated over 2000 children in circus arts	“children”
McCaffery, 2011 [65]	Grey literature: report	Mixed-methods	20	3–19 y
McCaffery, 2012 [64]	Grey literature: report	Mixed-methods	12	“young people under 24 years” (personal communication)
McCaffery, 2014 [63]	Grey literature: report	Mixed-methods	11	“Year 10 students”
McCutcheon, 2003 [37]	Peer-reviewed: masters thesis	Case studies	Case 1: 60 Case 2: 300 Case 3: not described Case 4: various Case 5: 100	“school-aged children”
McGrath & Stevens, 2019 [47]	Peer-reviewed: journal article	Mixed-methods	Surveys: 23 Focus group: 54	8–14 y (10 y 0 m)
Miller et al., 2016 [48]	Peer-reviewed: journal article	Exploratory study imbedded in larger prospective, clustered, quasi-experimental design	26	5–16 y (8 y 2 m)
Momesso dos Santos et al., 2015 [49]	Peer-reviewed: journal article	Not described	60	9–11 y (10 y 0 m)
Neave et al., 2020 [50]	Peer-reviewed: journal article	Between subjects repeated measures design	89	9–12 y (10 y 0 m)
O’Donnell, n.d. [66]	Grey literature: report	Pre-post survey design	125	5–17 y
Ott, 2005 [38]	Peer-reviewed: PhD thesis	Qualitative	11	8–18 y (13 y 4 m)
Pompe, 2021 [30]	Peer-reviewed: bachelors thesis	Case study	Not described	7–12 y, 13–18 y, 18–24 y
Rappaport, 2014 [73]	Grey literature: conference poster	Qualitative	Group 1: 6 Group 2: 68	12–17 y
Rivard et al., 2010 [51]	Peer-reviewed: journal article	Case study	Not described	10–19 y
Rodger & Kennedy-Behr, 2017 [32]	Peer-reviewed: book	Review of the literature	Not described	‘children’
Sahli et al., 2013 [52]	Peer-reviewed: journal article	Not described	24	5–6 y
Sakzewski, Ziviani, & Boyd, 2011 [53]	Peer-reviewed: journal article	Secondary analysis of single-blind matched-pairs randomized comparison trial	61	5–16 y (10 y 2 m)
Sakzewski, Ziviani, Abbott, et al., 2011a [54]	Peer-reviewed: journal article	Single-blind matched-pairs randomized comparison trial	62	5–16 y (10 y 2 m)
Sakzewski, Ziviani, Abbott, et al., 2011b [55]	Peer-reviewed: journal article	Single-blind matched-pairs randomized comparison trial	62	5–16 y (10 y 2 m)
Sakzewski, Miller, et al., 2015 [56]	Peer-reviewed: journal article	Matched-pairs randomized comparison trial	25	5–16 y (8 y)
Sakzewski, Provan, et al., 2015 [57]	Peer-reviewed: journal article	Secondary analysis of two single-blind matched-pairs randomized comparison trials	82	5–16 y (Group 1: 10.2 y, Group 2: 8.7 y)
Sakzewski, Ziviani, et al., 2015 [16]	Peer-reviewed: book chapter	N/A	N/A	“Children”
Sakzewski et al., 2012 [60]	Peer-reviewed: journal article	Single-blind matched-pairs randomized comparison trial	63	5–16 y (10 y 2 m)
Sakzewski, Ziviani, Abbott, et al., 2011c [61]	Peer-reviewed: journal article	Single-blind matched-pairs randomized comparison trial	57	5–16 y (10 y 2 m)
Seay, 2004 [39]	Peer-reviewed: masters thesis	Not described	157	6th–8th Grade
Seymour, 2012 [40]	Peer-reviewed: masters thesis	Qualitative	Not described	“children”
Seymour & Wise, 2017 [58]	Peer-reviewed: journal article	Not described	Not described	3–5 y
Smith et al., 2017 [70]	Grey literature: report	Mixed-methods	11 circus programs	6–18 y
Spiegel et al., 2015 [59]	Peer-reviewed: journal article	Mixed methods case study	Not described	“youth and children”
Stevens et al., 2019 [14]	Peer-reviewed: journal article	Qualitative	55	8–14 y (10 y 0 m)
Taylor & Taylor, 2004 [62]	Peer-reviewed: journal article	Case study	20	12–18 y
Trotman, 2013a [67]	Grey literature: report	Mixed-methods	28	8–10 y
Trotman, 2013b [68]	Grey literature: report	Qualitative	13	0–4 y
Valentini et al., 2020 [74]	Grey literature: conference proceeding	Qualitative	211	9–12 y (10 y 0 m)
Van Es et al., 2021 [21]	Peer-reviewed: journal article	Qualitative	11	5–20 y

## Appendix E. Extracted Outcome Data

**Table A6:** Health & wellbeing outcomes and assessments.

Citations	For Whom	Outcome Measures & Time Point	Health & Wellbeing Outcomes Reported
Agans et al., 2019 [17]	General population (circus school)	Single timepoint in an ongoing program: quantitative survey. Autonomy and autonomy support Competence and competence support Relatedness and relatedness support Intrinsic motivation Concentration Positive and negative affect Positive youth development Grit	Circus provided psychological need satisfaction Children in circus group exhibited intrinsic motivation, concentration, grit, positive affect, and positive youth development, and low levels of negative affect Relatedness is a strong predictor of intrinsic motivation, positive and negative affect, and positive youth development
Barnett et al., 2020 [18]	General population (primary school)	Timepoint not described: literature review	Circus is compatible with the concept of physical literacy and incorporates learning in all four domains of physical literacy
Biquet, 2014 [35]	“living in a warzone”	Single timepoint in an ongoing program: participant observations, semi-structured interviews.	Theme 1: a constructive hobby (participant perseverance, respect, solidarity) Theme 2: a restoration of hope and self-confidence (participant self-esteem) Theme 3: a federating project (community goals, cooperation not competition, inclusion)
Bolton, 2004 [33]	General population (ex-pats & local community)	Timepoint not described: custom survey, interviews, observations.	Increased self-awareness Experience fun & happiness Developing caution, co-ordination, and co-operation Increased trust and confidence
Bonk, 2019 [34]	General population (circus school)	Single timepoint immediately following intervention: Body map artwork & participant interview.	Improved mood Improvements in kinaesthetic awareness, emotional regulation skills and communication
Boyd et al., 2010 [42]	Cerebral palsy: unilateral hemiplegia	Protocol: Data to be collected at 3 timepoints: prior to, on completion, and 26 weeks post- intervention. Melbourne Assessment of Unilateral Upper Limb Function (MUUL) Assisting Hand Assessment (AHA) Jebsen Taylor Test of Hand Function (JTTHF) Canadian Occupational Performance Measure (COPM) Assessment of Life Habits (LIFE-H) Children’s Assessment of Participation and Enjoyment (CAPE) School Function Assessment (SFA) Cerebral Palsy Quality of Life (CPQOL-Child) KIDSCREEN-52	Protocol only (no outcomes described)
Boyd et al., 2013 [41]	Cerebral palsy: unilateral hemiplegia	Protocol: Data to be collected at 3 timepoints: prior to, on completion, and 26 weeks post- intervention. MUUL (primary) AHA (primary) JTTHF (secondary) Box and Blocks Test (secondary) Children’s Hand-use Experience Questionnaire (secondary) LIFE-H (secondary) CPQOL-Child (secondary) Canadian Occupational Performance Measure (COPM) (secondary) Dimensions of Mastery Questionnaire (secondary) Paediatric Volitional Questionnaire (secondary)	Protocol only (no outcomes described)
Cadwell & Rooney, 2013 [71]	General population (circus school)	Single timepoint in an ongoing program: self-reporting questionnaires, panel discussion & survey ‘game’	Ceiling effect for positive emotional expression and activity (high majority of the participants reported happiness as being a primary response to circus) Significant relationship between reporting experiencing negative emotions and periods of inactivity during the circus activity
Caldwell, 1996 [75]	General population (circus school)	Timepoint not described: observation.	Increased perseverance Learning social skills A chance to shine
Candy, 2017 [20]	General population	Single timepoint in an ongoing program: custom quantitative surveys.	>50% of participants responses were “somewhat agree” or “agree” to improvements in strength, flexibility, circus skills, coordination, friendship, body awareness, confidence, cooperation 100% of participants replied “completely agree” to the statement “circus has helped me improve my happiness” Parents reported circus training is developing their child in a variety of fields
Cohen, 2018 [43]	General population (circus school)	Data collected at 3 timepoints (no further description provided): results from Smith, 2017	Large positive change in individual youths’ social-emotional behavioural skills (emotion management, teamwork, initiative, empathy, responsibility, problem solving)
Csuros, 2015 [44]	Children in psychiatric care: “conduct disorder, hyperactivity, attention deficit disorder (ADD), autism, spectrum disorders and psychiatric disorders requiring emergency care (crisis conditions, suicidal intensions or attempted suicide, self-destructive behaviour, sudden psychotic conditions), schizophrenia, mood disorders, anxiety disorders, post-traumatic disorder, emerging personality disorders, mood disorders, obsessive compulsive disorder, eating disorders, attachment disorders.”	Timepoint not described: observations.	Developing self-efficacy Increased perseverance and motivation Developing emotional regulation skills Improved cooperation with peers
Fernandez et al., 2018 [22]	Children with developmental difficulties (without a specified medical condition or disability).	Single timepoint, immediately following intervention: Paediatric Volitional Questionnaire (PVQ) V2.1 (used to triangulate qualitative data) field notes (incl. videos) semi-structured interview with child and parent/s.	Having fun, experiencing enjoyment Improved competence and confidence Developed social skills (making friends, sense of belonging) Improved physical skills and function, fitness, strength & skill development
Fournier et al., 2014 [4]	Children in psychiatric care: mood disorders, anxiety disorders, relational or behavioural problems, attention deficit hyperactivity disorder (ADHD), psychotic disorders, eating disorders.	Data collected at 3 timepoints: prior to, immediately following and 3-months after intervention. Semi-structured interviews with patients and clinicians 13 patients also completed custom questionnaires.	Clinicians reported substantial differences between time 1 and time 2 or 3; in the patient’s dependency, inactivity, social isolation, and psychiatric symptoms Patients reported no significant difference in their social skills, ease in relationships and self-esteem
Gilmore et al., 2010 [45]	Cerebral palsy	Single timepoint during intervention: Semi-structured interviews, video footage and recall diary.	Doing the camp: there was a strong sense of motivation and engagement associated with the circus activities, with the camp described as “fun” and “something different to usual therapy” Gains: children identified being able to perform activities at home more easily using both hands after attending the camp
Heller & Taglialatela, 2018 [6]	ADD, ADHD, anxiety, autism, behavioural challenges, impulse control challenges (sourced from personal communication with author).	Data collected at 3 timepoints: prior to intervention, end of first intervention block, end of second intervention block. Parental report using custom online surveys.	Improved physicality, teamwork and following directions Increased sociability and emotional control (non-significant)
Kiez, 2015 [36]	General population (primary school)	Data collected at 2 timepoints: prior to and on completion of intervention. PLAY Fun, an assessment of motor competence, confidence, and comprehension of 18 movement skills. PLAY Inventory, a self-reported checklist of participation in physical activity Play Self, a self-report of physical literacy.	Play Fun: Improved motor competence, increased confidence, and increased comprehension Play Inventory: increase in participation in physical activity Play Self: increased physical literacy
Kinnunen et al., 2013 [69]	Variety of populations, including “special needs” class (school); general population (school & pre-school); ADD/ ADHD, autism; children of families who use the child welfare services and are dealing with crises; children with visual impairment.	Single timepoint in an ongoing program: surveys and interviews.	More physically active, confident, persevered, learnt new skills. Improved group dynamics, improved mood, improved self-esteem, increased social activity Improved motor skills, body awareness, trust in parents, improved communication, connecting with others, joy, physical skills, family relationships, body control and mobility Improved physical stamina and muscle tone, fitness, body perception, quality of movement, mobility, and flexibility & spatial perception
Kriellaars et al., 2019 [8]	General population (primary school)	Data collected at 2 timepoints: prior to and on completion of intervention. PLAY Fun, an assessment of motor competence, confidence, and comprehension of 18 movement skills. PLAY Inventory, a self-reported checklist of participation in physical activity Play Self, a self-report of physical literacy.	Play Fun: Improved motor competence, increased confidence, and increased comprehension Play Inventory: increase in participation in physical activity Play Self: increased physical literacy
Kovalenko, 2018 [29]	“at risk”: children from difficult family circumstances, adolescents who have committed an offence, children with disability.	Data collected over a 12-month period in an ongoing program: observation and interviews.	Improved communication, social skills, emotional regulation Acquired physical skills and improved physical fitness Improvements not noticeable at school or with family (limited crossover)
Loiselle et al., 2019 [46]	Young people with physical disabilities including: cerebral palsy, leukodystrophy, Steiner’s syndrome, Childhood callosotomy sequelae for uncontrolled epilepsy, Friedrich’s ataxia, traumatic acquired brain injury (ABI), severe burns in infancy	Data collected at 3 timepoints: prior to, mid-point and on completion of intervention. semi-structured interviews	Optimisation of communication skills, physical mobility, improved social connections, taking initiative, community participation Improved self-perception and confidence in physical, emotional, and cognitive capacities
Maglio & McKinstry, 2008 [9]	General population (school)	Single timepoint immediately following intervention: observations journal entries evaluation tool based on Victorian Essential Learning Standards (VELS) criteria.	Fun, motivating experience Increased positive risk taking both physically and emotionally Promotion of physical health and body awareness Skill acquisition Increased self-confidence and self-efficacy Improves social connectedness, teamwork, and leadership skills Experienced a sense of belonging
Mason, 2013 [72]	“living in a warzone”	Timepoint not described: observation	Decreased destructive behaviour Increase in amount of time able to focus and concentration Indications of increased trust Improved communication Increased ability to understand and follow instructions
McCaffery, 2011 [65]	School students with moderate to severe learning difficulties	Single timepoint immediately following intervention: custom survey interviews focus group	Increased self-confidence and self-esteem and physical well-being Improved communication skills Enjoyment and enthusiasm Improved social skills (taking turns, integration into a group) Acquisition of new skills
McCaffery, 2012 [64]	Autism	Single timepoint immediately following intervention: custom survey informal interviews observation	Improved confidence, self-belief, and fitness Improved social-emotional skills (displaying emotion and trusting other people)
McCaffery, 2014 [63]	“at risk” school students: at-risk of disengagement, poor attendance or leaving school early.	Single timepoint immediately following intervention: custom survey interviews	Improved attitude towards school and peers Improved engagement Improved confidence, communication, and concentration Acquisition of new skills Improvements in mood and increased positive behaviour Improved confidence and resilience
McCutcheon, 2003 [37]	“at risk” school students	Single timepoint in ongoing programs: Interviews and observation fieldwork collection of archival documents and artifacts	Physical benefits: increased physical fitness Psychological benefits: overall decrease in violent and anti-social behaviour in the school, the home and the community, improved community, and school-based relationships
McGrath & Stevens, 2019 [47]	General population (circus school)	Data collected pre- and post-intervention: KIDSCREEN-27 & focus group	Improvements in physical wellbeing and family relationships (not statistically significant) Improvements in resilience, socialisation, and mental wellbeing including stress relief, self-esteem, and confidence
Miller et al., 2016 [48]	Cerebral palsy: unilateral hemiplegia	Single timepoint: mid-intervention Dimensions of Mastery Questionnaire PVQ	Children in circus group were more motivated and engaged
Momesso dos Santos et al., 2015 [49]	Overweight	Single timepoint: Blood sample (lymphocyte, plasma glucose & insulin concentration, plasma inflammatory markers)	Improved immune system function Reduction in lymphocyte proliferation
Neave et al., 2020 [50]	General population (school)	Data collected pre- and post-intervention: Youth Life Orientation Test (YLOT) Stirling Children’s Wellbeing Scale (SCWS) Physical Activity Questionnaire (PAQ-C) Strengths and Difficulties Questionnaire (SDQ)	Circus participants had fewer emotional problems (SDQ) (psychosomatic illness, worries, unhappiness, nervousness, lack of confidence and fearfulness), no other significant differences
O’Donnell, n.d. [66]	“at-risk” due to socioeconomic status, single-parent homes, and/or a history of truancy and disruptive classroom behaviour at school; inpatient psychiatric unit.	Data collected pre- and post-intervention: Kutcher Adolescent Depression Scale (KADS) Self-Efficacy Questionnaire for Children (SEQ) Individual Protective Factors Index (IPFI) The Life Orientation Test-Revised (LOT-R) Multidimensional Scale of Perceived Social Support (MSPSS) Rosenberg Self-Esteem Scale (RSES)	Improved levels of peer social support (t(60) = 2.04, *p* < 0.05), self-esteem (t(49) = 3.75, *p* < 0.001), and cooperation skills (t(60) = −2.46, *p* < 0.05) Improved but not statistically significant changes in Depression, Social Self-Efficacy, Self-Control, Optimism, or Suicidality. Increased physical activity and body control, improved group dynamics and cooperation, and better family dynamics
Ott, 2005 [38]	General population (circus school)	Data collected over a 5-month period and then followed up 4 and 5 years later: semi-structured interviews & observation	Developing interpersonal relationships and social skills Increased strength and sense of identity
Pompe, 2021 [30]	Inpatient psychiatric unit	Timepoint not described: observations & case studies	Strengthened body image through increased body awareness, and new movement skills An avenue for emotional processing and regulation Increased confidence and trust in body’s capabilities Improved social skills: turn-taking, peer-assisted learning, increasing trust
Rappaport, 2014 [73]	Inpatient psychiatric unit: mood disorder, bipolar, depression; deaf, hard of hearing, or othercommunication difficulties.	Timepoint not described: thematic analysis of program evaluations	Circus enabled participation in movement & exercise; enhanced positive affect, increased self-determination Improved focus & attention and social skills
Rivard et al., 2010 [51]	Vulnerable young people in “difficult situations”.	Single timepoint in an ongoing program: observation and interviews	Improved body awareness, fitness, skill development Increased self-esteem
Rodger & Kennedy-Behr, 2017 [32]	Cerebral palsy	Timepoint not described: literature review drawing on results from Sakzewski et al. 2011 a [54], b [55], c [61]	Promotes autonomy and enhances motivation Provides opportunities for motor and social skill development Encourages persistence and self-confidence
Sahli et al., 2013 [52]	General population (circus school)	Single timepoint in an ongoing program: force platform centre of pressure excursions for: static-eyes open (S-EO) static-eyes closed (S-EC) dynamic-ML-eyes open (D-ML-EO) dynamic-ML-eyes closed (D-ML-EC) dynamic-AP-eyes open (D-AP-EO) dynamic-AP-eyes closed (D-AP-EC).	Improved postural control
Sakzewski, Ziviani, & Boyd, 2011 [53]	Cerebral palsy: unilateral hemiplegia	Data collected at 3 timepoints: prior to, on completion, and 26 weeks post- intervention. MUUL AHA COPM	Children with poorer hand function as baseline benefitted most from the intervention Improved bi-manual performance was associated with increased movement efficiency
Sakzewski, Ziviani, Abbott, et al., 2011a [54]	Cerebral palsy: unilateral hemiplegia	Data collected at 3 timepoints: prior to, on completion, and 26 weeks post- intervention. MUUL (primary) AHA (primary) grip strength (secondary) 2-point discrimination test (secondary) JTTHF (secondary)	Significant improvements in bi-manual performance following intervention. Small difference between training approaches (both including circus), with one approach (BIM) showing retained gains at 26 weeks, and the other (CIMT) showing additional improvements in unimanual capacity
Sakzewski, Ziviani, Abbott, et al., 2011b [55]	Cerebral palsy: unilateral hemiplegia	Data collected at 3 timepoints: prior to, on completion, and 26 weeks post- intervention. COPM (primary) LIFE-H (secondary) Children’s Assessment of Participation and Enjoyment (CAPE) (secondary) School Function Assessment (SFA) (secondary)	Significant gains in occupational performance for both groups that were retained at 26 weeks Significant improvements in self-care domain of LIFE-H Changes in participation were evident and related to goals
Sakzewski, Miller, et al., 2015 [56]	Cerebral palsy: unilateral hemiplegia	Data collected at 3 timepoints: prior to, on completion, and 26 weeks post- intervention. MUUL (primary) AHA (primary) COPM (secondary) JTTHF (secondary) Box and Blocks Test (secondary) Children’s Hand Use Experience Questionnaire (secondary)	Increased satisfaction and performance of COPM goals Improved dexterity of the upper limb Significant improvements on AHA Some children had a significant improvement on the MUUL
Sakzewski, Provan, et al., 2015 [57]	Cerebral palsy: unilateral hemiplegia	Data collected at 3 timepoints: prior to, on completion, and 26 weeks post- intervention. MUUL (primary) AHA (primary) COPM (secondary) JTTHF (secondary)	Higher dosages result in greater improvements in bimanual performance, dexterity and quality of movement Improved occupational performance
Sakzewski, Ziviani, et al., 2015 [16]	Cerebral palsy: unilateral hemiplegia	Literature review in book chapter	Circus activities can support psychological need satisfaction
Sakzewski et al., 2012 [60]	Cerebral palsy: unilateral hemiplegia	Data collected at 3 timepoints: prior to, on completion, and 26 weeks post- intervention. Cerebral Palsy Quality of Life Assessment for Children (CPQOL-Child; self-report and parent proxy versions) (primary) KIDSCREEN-52 (secondary)	Both groups improved quality of life, and these changes were retained at 3-months
Sakzewski, Ziviani, Abbott, et al., 2011c [61]	Cerebral palsy: unilateral hemiplegia	52-week follow-up (single time point) MUUL	Gains in unimanual capacity, bimanual performance and individual outcomes are retained at 1-year
Seay, 2004 [39]	General population (school)	Data collected at 3 timepoints: prior to, on completion, and 3-month post- intervention. Rosenberg Self-Esteem Scale (RSES) Revised Body-Esteem Scale Nowicki-Strickland Internal-External Locus of Control Scale (NSLCS) for Children and Adolescents Physical Activity Motivation Scale Custom questionnaires created to assess social involvement and participation were used.	No significant difference in increased psychosocial functioning significant increase in parent’s rating of social activity and participation compared to control group
Seymour, 2012 [40]	Autism and other neurodiversities	Timepoint not described observations interviews	Participants develop trust and community, physical development and personal confidence, improvements in day-to-day tasks, improvements in muscle tone and motor coordination; improvement in posture; improved attention; improved ability to participate in a group and confidence in social interactions; improved self-perception and motivation; improved coordination, and attention regulation
Seymour & Wise, 2017 [58]	Autism	Timepoint not described observations interviews	Circus builds trust & connection, belonging, coordination, spatial awareness, joy, develops body awareness and fitness, improves confidence and trust in physical capabilities
Smith et al., 2017 [70]	General population (circus school)	Data collected at baseline & completion of 3 programs over 3 timepoints: observation custom surveys	Participants demonstrated positive and substantial social-emotional behavioural skill growth
Spiegel et al., 2015 [59]	Youth “living on the street”	Protocol—timepoint not described: observation focus groups, interviews, and questionnaire surveys photovoice	From included literature review: circus promotes creativity and building perseverance, team building, social engagement, and social inclusion
Stevens et al., 2019 [14]	General population (circus school)	Single timepoint in ongoing program: focus group	Improvements in mental well-being (relief from stress, built self-esteem and confidence) Enjoyment of physical activity Aided socialisation skills Built resilience
Taylor & Taylor, 2004 [62]	Range of intellectual and/or physical disabilities (mild to severe)	Timepoint not described: observation of videos of practice & performance	Enhanced communication and physical skills, Improved self-esteem and self-confidence and positive changes in behaviour patterns
Trotman, 2013a [67]	General population (school)	Single timepoint following intervention: ratings by the children against a range of elements such as coordination and confidence interviews with teacher and parents	Half of the 19 children who responded said that their communication with others had improved and around a third stated that their confidence, coordination, concentration, comfort in a group and balance had improved Teacher reports increased confidence, improved coordination and motor skills, enhanced teamwork and peer relationships Parents report increased confidence, having fun, trying new things, being involved and a part of something
Trotman, 2013b [68]	General population (circus school)	Single timepoint in ongoing program: interviews and custom survey.	Increased confidence and willingness to try new things, Developing socialisation and trust building, Moderation of difficult behaviour, managed risk taking and positive and fun interaction between children and adults Learnings around taking turns, cooperating, and following instructions Increased confidence and willingness to try new things Improved physical skills
Valentini et al., 2020 [74]	General population (primary school)	Timepoint not described: literature review	Circus provides opportunities to develop motor skills, dexterity, concentration, self-esteem
Van Es et al., 2021 [21]	Refugee youth/ children and youth with traumatic war experiences.	Single timepoint during intervention: Semi-structured interviews and participant observations	Participants increased physical activity, while impulsive behaviour decreased. Experiencing success and feelings of competence through visible progress improved self-esteem Increased interpersonal trust Opportunities for social interaction and building relationships, may also promote social support Preliminary evidence of enhanced resilience in refugee youth
